# High local failure rates despite high margin‐negative resection rates in a cohort of borderline resectable and locally advanced pancreatic cancer patients treated with stereotactic body radiation therapy following multi‐agent chemotherapy

**DOI:** 10.1002/cam4.4527

**Published:** 2022-02-10

**Authors:** Colin Hill, Shuchi Sehgal, Wei Fu, Chen Hu, Abhinav Reddy, Elizabeth Thompson, Amy Hacker‐Prietz, Dung Le, Ana De Jesus‐Acosta, Valerie Lee, Lei Zheng, Daniel A. Laheru, William Burns, Matthew Weiss, Christopher Wolfgang, Jin He, Joseph M. Herman, Jeffrey Meyer, Amol Narang

**Affiliations:** ^1^ Department of Radiation Oncology and Molecular Radiation Sciences Johns Hopkins University School of Medicine Baltimore Maryland USA; ^2^ Philadelphia College of Osteopathic Medicine Philadelphia Pennsylvania USA; ^3^ Department of Biostatistics and Bioinformatics Johns Hopkins University School of Medicine Baltimore Maryland USA; ^4^ Department of Pathology Johns Hopkins University School of Medicine Baltimore Maryland USA; ^5^ Department of Medical Oncology The Sidney Kimmel Comprehensive Cancer Center Bloomberg‐Kimmel Institute for Cancer Immunotherapy at Johns Hopkins Baltimore Maryland USA; ^6^ Department of Surgery Johns Hopkins University School of Medicine Baltimore Maryland USA; ^7^ Department of Surgery Zucker School of Medicine at Hofstra/Northwell Lake Success New York USA; ^8^ Department of Surgery New York University Grossman School of Medicine New York New York USA

**Keywords:** BRPC, LAPC, locoregional failure, multi‐agent CTX, PDAC, SBRT

## Abstract

**Background:**

Stereotactic body radiation therapy (SBRT) for patients with borderline resectable and locally advanced pancreatic adenocarcinoma (BRPC/LAPC) remains controversial. Herein, we report on surgical, pathologic, and survival outcomes in BRPC/LAPC patients treated at a high‐volume institution with induction chemotherapy (CTX) followed by 5‐fraction SBRT.

**Methods:**

BRPC/LAPC patients treated between 2016 and 2019 were retrospectively reviewed. Surgical and pathological outcomes were descriptively characterized. Overall survival (OS) and progression‐free survival (PFS) were analyzed using Cox proportional hazard regression. Locoregional failure and distant failure were analyzed with Fine–Gray competing risk model.

**Results:**

Of 155 patients, 91 (59%) had LAPC and 64 (41%) had BRPC. Almost all were treated with induction multi‐agent CTX with either FOLFIRINOX (75%) or gemcitabine and nab‐paclitaxel (24%) for a median duration of 4.0 months (1–18 months). All received SBRT to a median dose of 33 Gy. Among 64 BRPC patients, 50 (78%) underwent resection, of whom 48 (96%) achieved margin‐negative (R0) resection. Among 91 LAPC patients, 57 (63%) underwent resection, of whom 50 (88%) achieved R0 resection. Despite the high R0 rate, 33% of patients experienced locoregional failure, which was a component of 44% of all failures. After SBRT, median OS and PFS were 18.7 and 7.7 months, respectively. After SBRT, 1‐ and 2‐year OS probabilities were 70% and 45%, whereas, from diagnosis, they were 93% and 51%.

**Conclusions:**

Although a high proportion of BRPC/LAPC patients treated with induction multi‐agent CTX followed by SBRT successfully achieved R0 resection, locoregional failure remained common, highlighting the need to continue to optimize radiation delivery in this context.

## Graphical abstract

A high proportion of patients in a modern cohort of BRPC/LAPC patients treated with multi‐agent CTX followed by SBRT became eligible for surgical exploration and resection with 92% achieving a margin‐negative resection. Despite the high rate of R0 resection, 33% of patients experienced locoregional failure, and 44% of all failures included locoregional failure as a component, highlighting the need to continue to optimize radiation delivery in this context.

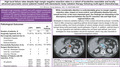

## 1 INTRODUCTION

Despite therapeutic advancements over the last decade, pancreatic adenocarcinoma (PDAC) remains an aggressive malignancy with dismal long‐term survival outcomes. By 2030, PDAC is expected to become the second leading cause of cancer‐related mortality.^1^, ^2^ Poor outcomes are driven by advanced presentation of disease and aggressive disease biology, with roughly half of patients presenting with evidence of metastatic disease at diagnosis. In patients with localized disease, the majority have tumors with extra‐pancreatic extension and involvement of key peri‐pancreatic vasculature, creating a significant barrier to complete surgical resection.

The role of radiation for localized pancreatic cancer remains controversial. In the locally advanced pancreatic cancer (LAPC) setting, radiation can be administered with the intent of improving local progression‐free survival and preventing associated impact on morbidity and mortality.^3^, ^4^, ^5^ Given the recognition that a much higher proportion of LAPC patients can undergo complete resection after upfront nonoperative therapy as compared to historical data, radiation can also be administered with the intent of margin sterilization and local recurrence risk reduction.^6^, ^7^, ^8^, ^9^, ^10^, ^11^, ^12^ Certainly, in the borderline resectable pancreatic cancer (BRPC) setting, margin sterilization and local recurrence risk reduction represent the primary goals of preoperative radiation therapy, with multiple studies, including two randomized controlled studies, suggesting benefit in this regard.^13^, ^14^, ^15^, ^16^, ^17^ While the referenced studies demonstrated encouraging outcomes with the use of preoperative radiation for BRPC, the recently presented Alliance A021501 randomized controlled trial did not show an additive benefit of radiation beyond neoadjuvant multi‐agent chemotherapy alone for BRPC.^18^

Given the discrepancy in these findings, we herein present operative and survival outcomes in a large cohort of BRPC/LAPC patients who were treated with stereotactic body radiation therapy (SBRT) at a single high‐volume institution. Multiple prior studies have reported on outcomes with SBRT for localized pancreatic cancer but are limited by small patient numbers, lack of modern systemic regimens such as FOLFIRINOX (FFX) or gemcitabine and nab‐paclitaxel (GnP), and use of SBRT for strictly definitive as opposed to preoperative intent.^6^, ^7^, ^8^, ^9^, ^10^, ^11^, ^12^, ^14^, ^15^, ^19^, ^20^, ^21^ As such, our intent is to demonstrate encouraging outcomes with the use of preoperative SBRT for resected BRPC/LAPC with respect to margin sterilization, but also to highlight opportunities for future refinement of the use of radiation therapy to increase durable local control in this setting.

## 2 METHODS

### 2.1 Patient population

With institutional review board approval and no ethical conflict of interest, all patients who were diagnosed with localized pancreatic cancer between 2016 and 2019 and who were treated at our institution with SBRT after induction chemotherapy were retrospectively reviewed. Patients were eligible for inclusion if they met the following study criteria: (1) histologic diagnosis of pancreatic adenocarcinoma; (2) BRPC or LAPC staging as per the National Comprehensive Cancer Network (NCCN) guidelines^22^; (3) treatment with SBRT following induction systemic therapy; and (4) sufficient follow‐up defined as ≥3 clinical encounters following SBRT.

### 2.2 Treatment course

Systemic therapy was prescribed at the discretion of the treating medical oncologist. In general, however, patients with a good performance status received multi‐agent chemotherapy, usually either modified FFX (mFFX) or gemcitabine and nab‐paclitaxel (GnP). During induction chemotherapy, patients were serially examined at approximately 3‐month intervals with a pancreatic protocol computed tomography scan to assess response. After completion of induction systemic therapy, patients with BRPC or LAPC were generally recommended to undergo SBRT at our institution, with re‐evaluation for surgical exploration after completion of SBRT. Prior to SBRT, patients underwent endoscopic fiducial placement under ultrasound guidance. At simulation, patients underwent a computed tomography scan with intravenous contrast and immobilization using a Vac‐Lok (CIVCO Medical Solutions, Coralville, IA, USA) or Alpha cradle (CIVCO Medical Solutions, Coralville, IA, USA) in the supine position with arms up. The SBRT course consisted of five fractions delivered over consecutive weekdays. Motion management was most commonly addressed using active breathing control (ABC, Elekta, Stockholm, Sweden), although a minority of patients were treated under a free‐breathing approach using a customized internal tumor volume expansion based on assessment with a four‐dimensional computed tomography scan. The clinical tumor volume (CTV) included gross disease as well as the full circumference of involved vasculature at the level of involvement. Planning tumor volume (PTV) was generated by applying a 2‐mm isotropic margin to the CTV, if a breath‐hold approach was utilized, or the iCTV, if a free‐breathing approach was utilized. Daily image guidance was utilized with both pre‐treatment and intra‐fraction cone beam computed tomography (CBCT) imaging. For pre‐treatment set‐up, patients were initially aligned to spine, with a subsequent translational shift applied to align to fiducials. Intra‐fraction variation that was noted on the intra‐fraction CBCT and that was greater than the PTV margin was also corrected.

Patients who were potential candidates for surgical exploration were generally restaged with computed tomography imaging between 4 and 6 weeks after the end of SBRT. For those patients that underwent resection, the resection specimens were processed per standard institutional grossing protocols. Specimen margins were identified using anatomic landmarks and orienting stitches by surgeon and were submitted for intraoperative frozen section and/or permanent section as per surgeon request. Pancreatic neck (parenchymal), common bile duct, and vascular margins, as applicable, were taken as shave sections. Uncinate margins, where applicable, were inked and then taken as perpendicular sections. Additional final and separate retroperitoneal/SMA margins were submitted separately per surgeon’s discretion. Shave margins were considered positive when tumor was present anywhere on the margin section. Perpendicular margin was considered positive when tumor was present at ink and distance to margin was noted when <1 mm. If unoriented, additional margins submitted separately by the surgeon were treated as shave margins. If oriented, the true margin was inked and the margin was sectioned perpendicular to the ink and treated as a perpendicular margin as described above.

Adjuvant or maintenance chemotherapy was at the discretion of the treating medical oncologist. After surgery for exploratory candidates or after SBRT for nonoperative candidates, patients were subsequently followed with surveillance pancreatic protocol computed tomography scans, initially at 3‐month intervals, with subsequent spacing of scans at the discretion of the clinical team. Cancer antigen (CA) 19‐9 levels were also generally obtained at follow‐up visits but were also at the discretion of the clinical team.

### 2.3 Clinical and pathological outcomes

Baseline demographics such as age, gender, performance status, stage, tumor location, tumor grade at biopsy, and carbohydrate antigen (CA) 19‐9 levels were recorded. Regarding treatment variables, type and duration of induction and adjuvant chemotherapy were recorded, as was radiation prescription dose. Surgical outcomes, including successful gross resection, margin status, nodal status, and pathological complete response (PCR; defined as no residual tumor), were reported with descriptive statistics.

### 2.4 Statistical analysis

Overall survival was recorded as the time from SBRT to death. The date of death was sourced from medical records and Social Security Death Index. If date of death was unavailable, survival was censored at the date of the last recorded clinical encounter. Progression‐free survival (PFS) was measured as the interval from the end of SBRT to the time of the first radiographic evidence of failure or death, whichever occurred earlier, and censored at the last date of recorded imaging follow‐up. Local progression (LP) and distant metastases (DM) were recorded as the time for first occurrence of locoregional or distant failure, respectively, or death, whichever occurred earlier. Locoregional failure included disease recurrence occurring within the surgical bed, extra‐pancreatic perineural tracts, regional nodal basins, or pancreatic remnant. OS and PFS were analyzed using the Kaplan–Meier method, and cumulative incidence of LP and DM was estimated. The association of PFS and OS with patient characteristics was assessed using univariate analysis (UVA) and multivariable analysis (MVA) via Cox proportional hazards models. Univariate and multivariable Fine‐Gray competing risk models with death as competing events were used to study the association between failure outcome and patient characteristics. Alive patients without observed events will be censored at the date of the last follow‐up. Only variables with *p* value less than 0.05 in UVA were selected into the MVA. Statistical analyses were performed using R 4.0.1.^23^

## 3 RESULTS

### 3.1 Clinical demographics and treatment characteristics

At our institution, 155 patients meeting inclusion criteria were retrospectively reviewed, including 91 patients (59%) with LAPC and 64 patients (41%) with BRPC. Clinical, demographic, and treatment characteristics are summarized in Table 1. Almost all patients had an Eastern Cooperative Oncology Group (ECOG) status of 0–1 (98%). Median CA 19‐9 at baseline prior to induction therapy was 215.2 U/mL (range: <1.0–7358.4 U/mL), with 57% of patients having a baseline CA 19‐9 greater than 90 U/mL. Induction FFX was administered to 116 (75%) patients, while 37 (24%) patients were treated with induction GnP. Median duration of induction chemotherapy before SBRT was 4.0 months (range: 1–18 months). Median CA 19‐9 after SBRT was 38.0 U/mL, with 30% of patients having a CA 19‐9 greater than 90 U/mL. Median SBRT dose was 33 Gy (range: 30–36 Gy) over five fractions, with 81% of patients being treated with ABC for motion management and 19% of patients being treated with a free‐breathing approach.

**TABLE 1:** Clinical demographics and treatment characteristics

	Total
Patients, *n*	155
Age (median, range)	66 (42–84)
Male gender (*n*, %)	80 (52)
ECOG PS (*n*, %)	
0	54 (35)
1	97 (63)
2	4 (2)
Tumor location (*n*, %)	
Head/neck/uncinated	108 (70)
Body/tail	47(30)
NCCN staging (*n*, %)	
BRPC	64 (41)
LAPC	91 (59)
Initial biopsy tumor grade (*n*, %)	
Poor	51 (40)
Moderate‐well	78 (60)
CA 19‐9 prior to SBRT (median)	215.2
CA 19‐9 < 90, *n* (%)	32 (36)
CA 19‐9 ≥ 90, *n* (%)	56 (64)
CA 19‐9 after SBRT (median)	38.0
CA 19‐9 < 90, *n* (%)	56 (70)
CA 19‐9 ≥ 90, *n* (%)	24 (30)
CT agent (*n*, %)	
FFX	116 (75)
GnP	37 (24)
Other	2 (1)
Induction CT duration, months (median, range)	4 (1–18)
SBRT dose, Gy (median, range)	33 (30–36)
Adjuvant therapy	
Received any CTX, *n* (%)	58 (37)
CTX duration, months (median, range)	2 (1–6)

Abbreviations: BRPC, borderline resectable pancreatic cancer; CA 19‐9, cancer antigen 19‐9; CT, chemotherapy; ECOG PS, Eastern Cooperative Oncology Group performance status; FFX, FOLFIRINOX; GnP, gemcitabine and nab‐paciltaxel; Gy, GrayLAPC: locally advanced pancreatic cancer; NCCN, National Comprehensive Cancer Network.

### 3.2 Operative and pathologic outcomes

After SBRT, 132 patients (85%) were eligible for surgical exploration. The median time between SBRT and exploration was 6.4 weeks (range: 1.6–25.2 weeks). Reasons for foregoing surgical exploration included imaging evidence of metastatic disease in 8 patients (5.2%), primary tumors that were too locally extensive in 10 patients (6.5%), and medical comorbidities that were prohibitive in 5 patients (3.2%). Of the 132 patients that were surgically explored, 107 patients (81%) were able to undergo gross total resection. Surgery was aborted in 18 patients (13.6%) due to intra‐operative findings of metastatic disease, extent of local disease in 5 patients (3.8%), and prohibitive fibrosis in 2 patients (1.5%). Of 107 patients who were resected, 98 patients (92%) achieved negative margins, 63 patients (59%) were node‐negative, and 8 patients (8%) achieved a pCR. Among 64 BRPC patients, 50 patients (78%) underwent resection, of whom 48 patients (96%) achieved margin‐negative resection. Among 91 LAPC patients, 57 patients (63%) underwent resection, of whom 50 patients (88%) achieved margin‐negative resection. These surgical outcomes are summarized in Table 2. Note that for 11 patients (11.2%), microscopic disease was present within 1 mm of the surgical margin. If such patients are included in the definition of margin‐positive resection, the margin‐negative resection rates for BPRC patients and LAPC patients, were 84% and 79%, respectively. Vascular reconstruction was required in 38 patients (36%) who were successfully resected. Vascular reconstruction frequency was comparable among resected BRPC (*n* = 17, 34%) and LAPC (*n* = 21, 37%) patients. After SBRT, 58 patients (37%) received adjuvant therapy for a median of 2 months (range: 1–6 months).

**TABLE 2:** Pathological outcomes

	LAPC	BRPC	All patients
Number of patients, *N*	91	64	155
Surgically explored, *N* (%)	74 (81)	58 (91)	132 (85)
Not surgically explored due to:			
Metastatic disease	5	3	8
Local extent	8	2	10
Medical reasons	4	1	5
Successfully resected, *N* (%)	57 (63)	50 (78)	107 (69)
Surgery aborted due to:			
Intra‐Op. metastatic disease	11	7	18
Intra‐Op. local extent	4	1	5
Intra‐Op. fibrosis	2	0	2
Number of patients with R0 resection, *N* (%)			
All patients	50/91 (55%)	48/64 (75%)	98/155 (63%)
Resected patients	50/57 (88%)	48/50 (96%)	98/107 (92%)

Abbreviations: BRPC, borderline resectable pancreatic cancer; F/U, Follow‐up; Intra‐Op, intra‐operative; LAPC, locally advanced pancreatic cancer; SBRT, stereotactic body radiation therapy.

### 3.3 Survival outcomes and patterns of failure

**FIGURE 1:**
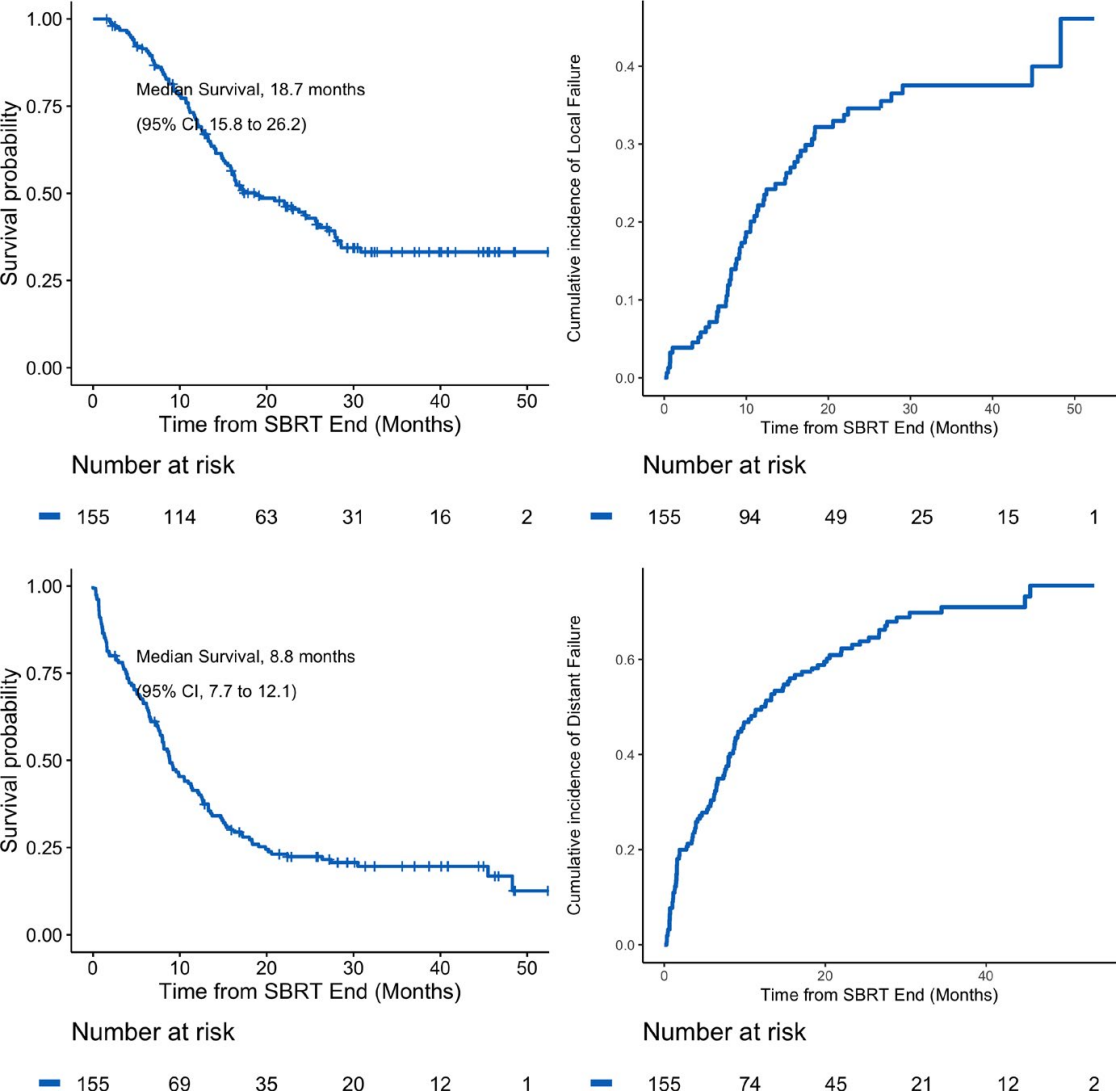
The Kaplan–Meier curves of (A) OS, (B) PFS and cumulative incidence functions of (C) LP, and (D) DM for the entire patient cohort from the end of SBRT

At last follow‐up, 92 patients had died. Median follow‐up from diagnosis for patients still alive was 38.8 months (12.2–59.1 months). Figure 1 illustrates survival outcomes for the cohort. From the end of SBRT, median overall survival (mOS) was 18.7 months (95% CI: 15.8–26.2 months), and the 1‐ and 2‐year probabilities of OS were 70% (95% CI: 63%–78%) and 45% (95% CI: 37%–54%), respectively. From diagnosis, mOS was 26 months (95% CI: 22–34 months), and the 1‐ and 2‐year probabilities of OS were 93% (95% CI: 90%–98%) and 51% (95% CI: 44%–60%), respectively. The median PFS after SBRT was 8.8 months (95% CI: 7.7–12.1 months), and the 1‐ and 2‐year probabilities of PFS were 41% (95% CI: 34%–50%) and 22% (95% CI: 17%–30%), respectively. Patterns of first failure on imaging follow‐up for all patients are summarized in Table 3 and included local failure in 21 patients (14%), distant failure in 64 patients (42%), and both local and distant failure in 30 patients (19%). As such, 33% of patients experienced local failure as a component of first failure, and 44% of all failures included local failure as a component. The 1‐ and 2‐year probabilities of local failure after SBRT were 22% (95% CI: 15%–29%) and 35% (95% CI: 27%–43%), respectively, whereas the 1‐ and 2‐year probabilities of distant failure were 49% (95% CI: 42%–57%) and 63% (95% CI: 55%–71%), respectively.

**TABLE 3:** Patterns of failure after SBRT

Type of first failure for cohort, *N* (%)	Total (*N* = 155)	*BRPC* (*N* = 64)	*LAPC* (*N* = 91*)*
Local failure	21 (14)	8 (13)	13 (14)
Distant failure	64 (42)	25 (39)	39 (43)
Synchronous failure	31 (19)	16 (25)	15 (17)

For those taken to surgery, the 1‐ and 2‐year LPFS probabilities after surgical resection were 70.9% and 54.2%, respectively, and median LPFS after resection was 27.7 months. Notably, 32 out of 98 patients (36%) with R0 resections recurred locally compared to 5 of 9 patients (56%) with R1 resections. If disease present within 1 mm of the margin was included in the definition of a margin‐positive resection, 12 of 20 patients (60%) with an R1 resection had evidence of local progression compared to 25 of 87 patients (29%) with an R0 resection. Patterns of failure for resected patients, stratified by pathologic features, are reported in Table S1. Among resected patients, there was no significant difference in LPFS on UVA based on key pathologic features (Table S2).

After SBRT, on multivariable analysis, poorly differentiated initial biopsy tumor grade, duration of neoadjuvant chemotherapy for less than 4 months, lack of adjuvant chemotherapy, and CA 19‐9 level ≥ 90 U/mL at any timepoint were significantly associated with inferior OS, whereas only the initial biopsy grade, lack of adjuvant chemotherapy, and CA 19‐9 ≥ 90 at any timepoint were associated with PFS (Table S3). Both initial biopsy grade and CA 19‐9 level ≥ 90 at any timepoint were associated with risk of distant metastasis. Complete resection was significantly associated with the risk of local progression. Although the association between surgical resection and OS did not meet statistical significance on MVA (*p* = 0.09), median OS after SBRT for resected patients was 27.1 months versus 10.0 months in unresected patients (*p* < 0.001), and 3‐year OS probabilities for resected and unresected patients was 43.7% (95% CI: 33.3%–54.3%) and 9.0% (95% CI: 0.0%–18.3%), respectively (Figure S1).

Among patients who underwent R0 resection, defined as disease at the margin, those who were treated with induction chemotherapy for ≥4 months as well as adjuvant chemotherapy (*n* = 42) trended toward better OS compared to remainder of the R0 cohort (Figure S2).

## 4 DISCUSSION

In a modern cohort of patients with BRPC/LAPC who were primarily treated with induction multi‐agent systemic therapy followed by SBRT, a high proportion of patients were able to undergo margin‐negative resection. Indeed, among BRPC patients, 75% of patients treated with SBRT and 96% of resected patients underwent margin‐negative resection. Even among LAPC patients, 55% of all patients treated with SBRT and 88% of resected patients underwent margin‐negative resection. Nevertheless, despite these high margin‐negative resection rates, local failure remained a common pattern of failure, with 33% of the cohort experiencing local failure as a component of first failure and with 44% of all failures including local failure as a component. This persistence of local failure highlights the need to continue to refine the administration of radiation to improve local control in this clinical setting.

The use of radiation therapy for BRPC remains highly debated. Initial creation of this classification was predicated on a high margin‐positive resection rate with upfront surgery in this sub‐population of patients. Multiple studies have now been published demonstrating high margin‐negative resection rates in BRPC after neoadjuvant chemotherapy and radiation. A randomized controlled study from Korea investigated upfront chemoradiation versus upfront surgery in BRPC patients and was terminated early due to a much higher rate of margin sterilization in the chemoradiation arm.^16^ Similarly, the PREOPANC study randomized both resectable and borderline resectable patients to upfront surgery versus preoperative gemcitabine‐based chemoradiation.^17^ Although the PREOPANC study was negative for the primary endpoint of overall survival, a dramatic difference was seen with respect to margin sterilization in favor of the chemoradiation arm (70% vs. 40%, *p* < 0.001).^17^ More importantly, significant improvement was also seen in local failure‐free interval and disease‐free survival.^17^ Even more, in the subset of patients with BRPC, overall survival in fact was improved with preoperative chemoradiation as compared to upfront surgery.^17^

However, the value of preoperative radiation in the setting of modern intensive neoadjuvant multi‐agent systemic therapy such as FFX remains controversial. This question has been explored in two studies led by the Alliance consortium. The first, Alliance A021101, was a small single arm study, in which BRPC patients were treated with FFX followed by chemoradiation.^13^ Of the 22 patients enrolled in this study, 15 (68%) underwent resection and 14 of 15 (93%) were resected with negative margins.^13^ The subsequent study, the Alliance 021501, was designed in a randomized fashion to explore the additive value of radiation beyond neoadjuvant FFX alone.^18^ This study incorporated hypo‐fractionated radiation based on prior institutional studies demonstrating efficacy with SBRT in this context.^10^, ^15^ Of the 40 patients who received radiation in A021501, only 19 (48%) underwent resection and only 14 (74% of resected patients, 35% of all patients treated with radiation) underwent margin‐negative resection (abstract only).^18^ The discrepancy between the Alliance 021501 results and those seen in our cohort is highlighted by the fact that even LAPC patients in our cohort experienced higher margin‐negative resection rates as compared to the BRPC patients in the RT arm of Alliance 021501, but definitive conclusions are premature and should await the final publication of this study.

Administration of radiation therapy for LAPC remains similarly contentious. Several historical randomized controlled trials have shown mixed results with the addition of either upfront or consolidative radiation to chemotherapy, but the antiquated radiation techniques and chemotherapeutic agents administered render these studies inapplicable to modern‐day practice.^24^, ^25^, ^26^, ^27^ The most relevant randomized controlled trial that asked this question was LAP07, in which LAPC patients were treated with induction gemcitabine for four cycles, and those patients without progression were subsequently randomized to two additional cycles of gemcitabine or consolidative chemoradiation.^3^ Although LAP07 was a negative study for the primary endpoint of OS, patients in the chemoradiation arm did experience significant improvement in local control.^3^ Importantly, systemic control was poor with gemcitabine alone, as 40% of patients were ineligible for randomization to chemoradiation due to progression.^3^ Furthermore, <5% of patients were surgically explored, preventing assessment of the role of radiation as a preoperative tool to aid in margin sterilization and local recurrence risk reduction.^3^ However, since the publication of LAP07, several reports from high‐volume institutions, including ours, have demonstrated much higher rates of complete resection in the setting of multi‐agent chemotherapy regimens such as FOLFIRINOX, with a corresponding improvement in OS in resected patients as compared to historical outcomes for LAPC patients.^8^, ^9^, ^10^, ^11^, ^12^ Notably, in these studies, radiation has been nearly universally incorporated in the preoperative regimen. Indeed, there is little precedent demonstrating similarly high rates of margin‐negative resection after multi‐agent systemic therapy alone, without radiation therapy, in LAPC patients.

**FIGURE 2:**
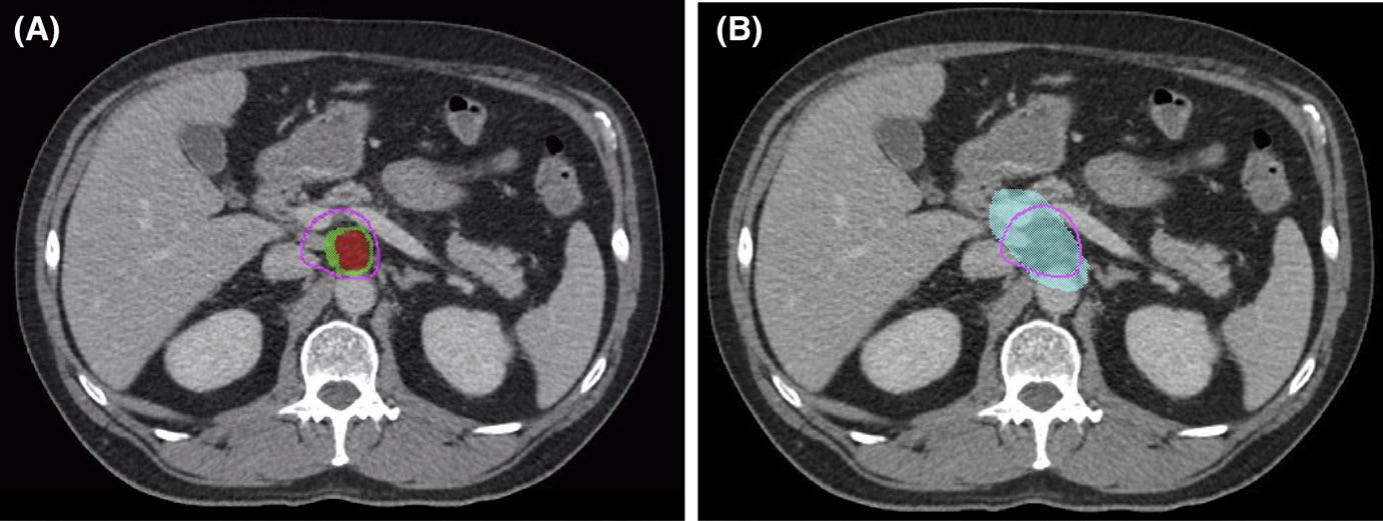
Axial view of planning computed tomography scan for a pancreatic cancer patient in the study. (A) In total, 33 Gy isodose line (purple line) is optimized to the gross tumor volume (GTV; red) and the planning tumor volume (purple) with the GTV encompassing all of the gross tissue and the tumor–vessel interface (TVI). (B) The triangle volume (light blue) encompasses all areas potentially at risk of microscopic disease spread beyond the level of the GTV based on extra‐pancreatic neural tracts and the 33 Gy isodose line (purple) from the original plan on the left is superimposed on the triangle to demonstrate the potential risk of undercoverage when optimizing plans only to the GTV and TVI

While considerable attention is understandably given to margin‐negative resection rates, impact on local control, and ultimately disease‐free and overall survival, is the more important goal. Our findings are interesting in the discrepancy observed between the low R1 resection rate and high local‐regional failure rate. Better understanding of the biological and anatomical drivers of local failure and the manner in which radiation can be refined to further improve local control should be pursued. Currently, there is little consensus regarding optimal clinical target volume design in the preoperative setting, with studies variably targeting gross disease only, gross disease and the full circumference of involved vasculature, and gross disease and more extensive elective tissue at risk, although how such elective tissue at risk should be defined remains unclear. In the surgical literature, much attention recently has been given to surgical clearance of the “triangle” of tissue that exists between the celiac artery, superior mesenteric artery, common hepatic artery, and portal vein and that contains a fat space with high density of at risk perineural tracts and lymphovascular channels.^28^, ^29^, ^30^ It stands to follow that such principles could apply to radiation field design. In fact, some data suggest that extra‐pancreatic perineural invasion is highly associated with local failure, and so characterization and coverage of such extra‐pancreatic perineural tracts may help to inform optimal target volume design.^31^, ^32^, ^33^ Figure 2 illustrates the difference in radiation field design between coverage solely of gross disease and involved vasculature versus elective coverage of the aforementioned “Triangle.” Certainly, optimal prescription dose in both the preoperative and definitive setting has also not been well defined, with recent data suggesting improved outcomes with dose‐escalation.^34^, ^35^ Given these data, multiple studies are underway exploring dose‐escalated radiation for BRPC/LAPC, although the proximity of stomach and small bowel render coverage with ablative dosing challenging.^36^, ^37^, ^38^ As such, exploration of novel strategies for intensifying dose delivery in this population should be pursued.^39^, ^40^ In addition, combined modality approaches that may help address pathways of therapeutic resistance to radiation should also be explored.^41^

Limitations inherent to our study include its retrospective, single‐arm, and single‐institution nature. Furthermore, with respect to surgical margins, it remains a matter of debate how specimens are processed and analyzed, and it remains unclear what constitutes a truly positive margin especially in a population that has received neoadjuvant therapy.^42^, ^43^ Additionally, we were unable to account for potential selection bias, as patients receiving radiation may represent a sub‐population of patients who did not progress on chemotherapy and therefore inherently may have better biology. Nonetheless, we believe these data to be an important demonstration of outcomes from a high‐volume pancreatic center.

## 5 CONCLUSION

In a modern cohort of BRPC/LAPC patients treated with chemotherapy followed by SBRT, a high proportion of patients were successfully resected with favorable pathologic outcomes. Despite this, a significant rate of local recurrence persists, highlighting the need to further optimize radiation dose delivery in this setting.

## ETHICS STATEMENT

This study was approved by the institutional review board.

## CONFLICT OF INTEREST

No significant conflict of interest to disclose for this manuscript.

## AUTHOR CONTRIBUTION

Colin Hill and Amol Narang were involved in conception and design, acquisition of data, analysis and interpretation of data and drafting the manuscript. Shuchi Sehgal was involved in acquisition of data. Wei Fu and Chen Hu were involved in analysis and interpretation of data. Abhinav Reddy was involved in analysis of data. Elizabeth Thompson was involved in interpretation of data and revision of the manuscript. Amy Hacker‐Prietz, Dung Le, Ana De Jesus‐Acosta, Valerie Lee, Lei Zheng, Daniel Laheru, William Burns, Matthew Weiss, Christopher Wolfgang, and Jin He were involved in drafting and revision of the manuscript. Joseph Herman and Jeffrey Meyer were involved in analysis and interpretation of data, and drafting and revision of the manuscript.

## Supporting information

Table S1

Table S2

Table S3

Fig S1

Fig S2

## Data Availability

All data generated and analyzed during this study are included in this published article (and its supplementary information files).
