# Supplementary material for: High local failure rates despite high margin‐negative resection rates in a cohort of borderline resectable and locally advanced pancreatic cancer patients treated with stereotactic body radiation therapy following multi‐agent chemotherapy
Source: Cancer Med. 2022 Feb 10;11(7):1659–68. doi: 10.1002/cam4.4527 (PMC8986142; doi:10.1002/cam4.4527)
Supplement: Supplementary file 1 — Table S1 [file CAM4-11-1659-s004.docx]

| **Supplementary Table 1:** Multivariable Analysis for Survival Outcomes after SBRT | | | | | | | | | |
| --- | --- | --- | --- | --- | --- | --- | --- | --- | --- |
|  | **OS** | | **PFS** | | **LP** | | | **DM** | |
| **Variable** | HR (95% CI) | *p*-value | HR (95% CI) | *p*-value | sHR (95% CI) | *p*-value | sHR (95% CI) | | *p*-value |
| **Baseline ECOG** |  |  |  |  |  |  |  | |  |
| 0 | 1 |  | 1 |  | 1 |  | 1 | |  |
| 1-2 | 1.45 (0.89-2.44) | 0.129 | NA |  | NA |  | NA | |  |
| **Initial Biopsy Tumor Grade** |  |  |  |  |  |  |  | |  |
| Poor | 1 |  | 1 |  | 1 |  | 1 | |  |
| Moderate - Well | 0.54 (0.33-0.89) | **0.014** | 0.62 (0.39-0.97) | 0.036 | NA |  | 0.60 (0.40-0.89) | | **0.0122** |
| **Duration. of Neoadjuvant CTX** |  |  |  |  |  |  |  | |  |
|  | 0.9 (0.76-0.97) | **0.016** | 0.92 (0.85-1.00) | 0.056 | NA |  | NA | |  |
| **Surgically Resected** |  |  |  |  |  |  |  | |  |
| No | 1 |  | 1 |  | 1 |  | 1 | |  |
| Yes | 0.62 (0.36-1.08) | 0.089 | 0.89 (0.52-1.53) | 0.679 | 2.07 (1.01-4.02) | **0.046** | NA | |  |
| **Adjuvant CTX** |  |  |  |  |  |  |  | |  |
| No | 1 |  | 1 |  | 1 |  | 1 | |  |
| Yes | 0.49 (0.27-0.87) | **0.014** | 0.51 (0.31-0.85) | 0.009 | NA |  | NA | |  |
| **CA 19-9 >=90** |  |  |  |  |  |  |  | |  |
| No | 1 |  | 1 |  | 1 |  | 1 | |  |
| Yes | 2.14 (1.21-3.78) | **0.008** | 2.67 (1.61-4.43) | 0.0001 | NA |  | 2.32 (1.49-3.61) | | **0.002** |

Abbreviations: OS: Overall Survival; PFS: Progression-Free Survival; LP: Local Progression; DM: Distant Metastases; HR: Hazard Ratio; sHR: Sub-distribution Hazard Ratio; ECOG: Eastern Cooperative Oncology Group; CTX: Chemotherapy; CA 19-9: Cancer Antigen 19-9
