# Supplementary material for: High local failure rates despite high margin‐negative resection rates in a cohort of borderline resectable and locally advanced pancreatic cancer patients treated with stereotactic body radiation therapy following multi‐agent chemotherapy
Source: Cancer Med. 2022 Feb 10;11(7):1659–68. doi: 10.1002/cam4.4527 (PMC8986142; doi:10.1002/cam4.4527)
Supplement: Supplementary file 3 — Table S3 [file CAM4-11-1659-s003.docx]

| **Supplementary Table 3:** Multivariable analysis of Pathological Outcomes for Local Progression Free-Survival from Surgery | | | | | |
| --- | --- | --- | --- | --- | --- |
| **Variable** | **Median LPFS (log-rank p)** | **UVA^**^** | | **MVA** | |
|  |  | HR (95% CI) | P* | HR (95% CI) | P^†^ |
| Pathologic Response (pCR vs no pCR) | Not reached vs 27.6 months (p=0.0711) | 0.190 (0.026-1.412) | 0.105 | 0.200 (0.027-1.497) | 0.117 |
| Pathologic Nodal status (pN0 vs pN+) | 47.4 vs 19.9 months (p=0.262) | 0.699 (0.372-1.313) | 0.266 |  |  |
| Lymphovascular invasion (LVI- vs LVI+) | 47.4 vs 19.9 months (p=0.211) | 0.645 (0.323-1.291) | 0.215 |  |  |
| Perineural invasion (PNI- vs PNI+) | 47.4 vs 19.9 months (p=0.442) | 0.760 (0.376-1.535) | 0.444 |  |  |
| Margins (R0 vs R1) | 47.4 vs 18.2 months (p=0.189) | 0.563 (0.236-1.344) | 0.196 | 0.623 (0.260-1.492) | 0.288 |

*Univariate Cox P value

**UVA P values <0.2 were selected for MVA

^†^Multivariable Cox P value
