# Supplementary figures and images for: High local failure rates despite high margin‐negative resection rates in a cohort of borderline resectable and locally advanced pancreatic cancer patients treated with stereotactic body radiation therapy following multi‐agent chemotherapy
Source: Cancer Med. 2022 Feb 10;11(7):1659–68. doi: 10.1002/cam4.4527 (PMC8986142; doi:10.1002/cam4.4527)

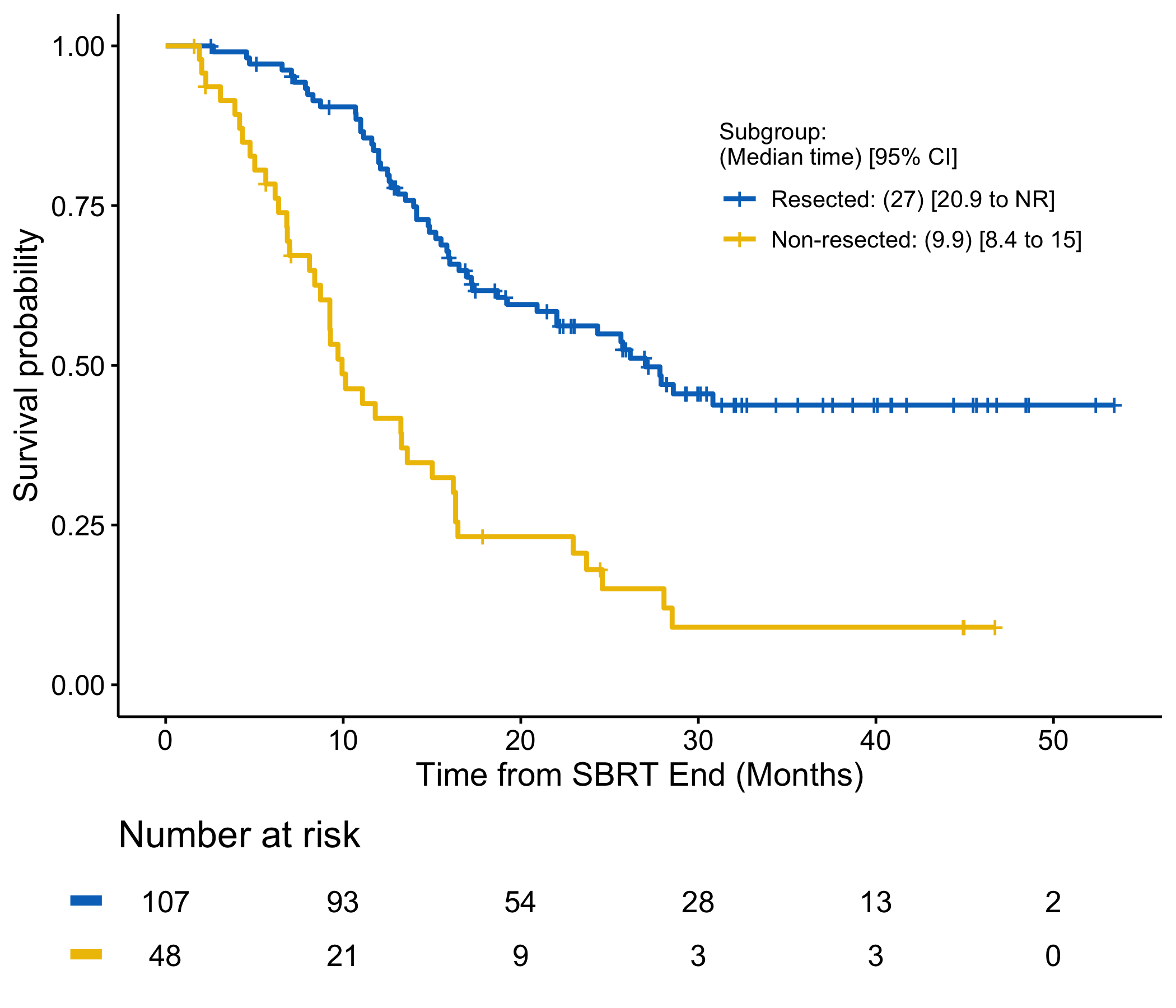

Supplement: Supplementary file 4 — Fig S1 [file CAM4-11-1659-s005.png]

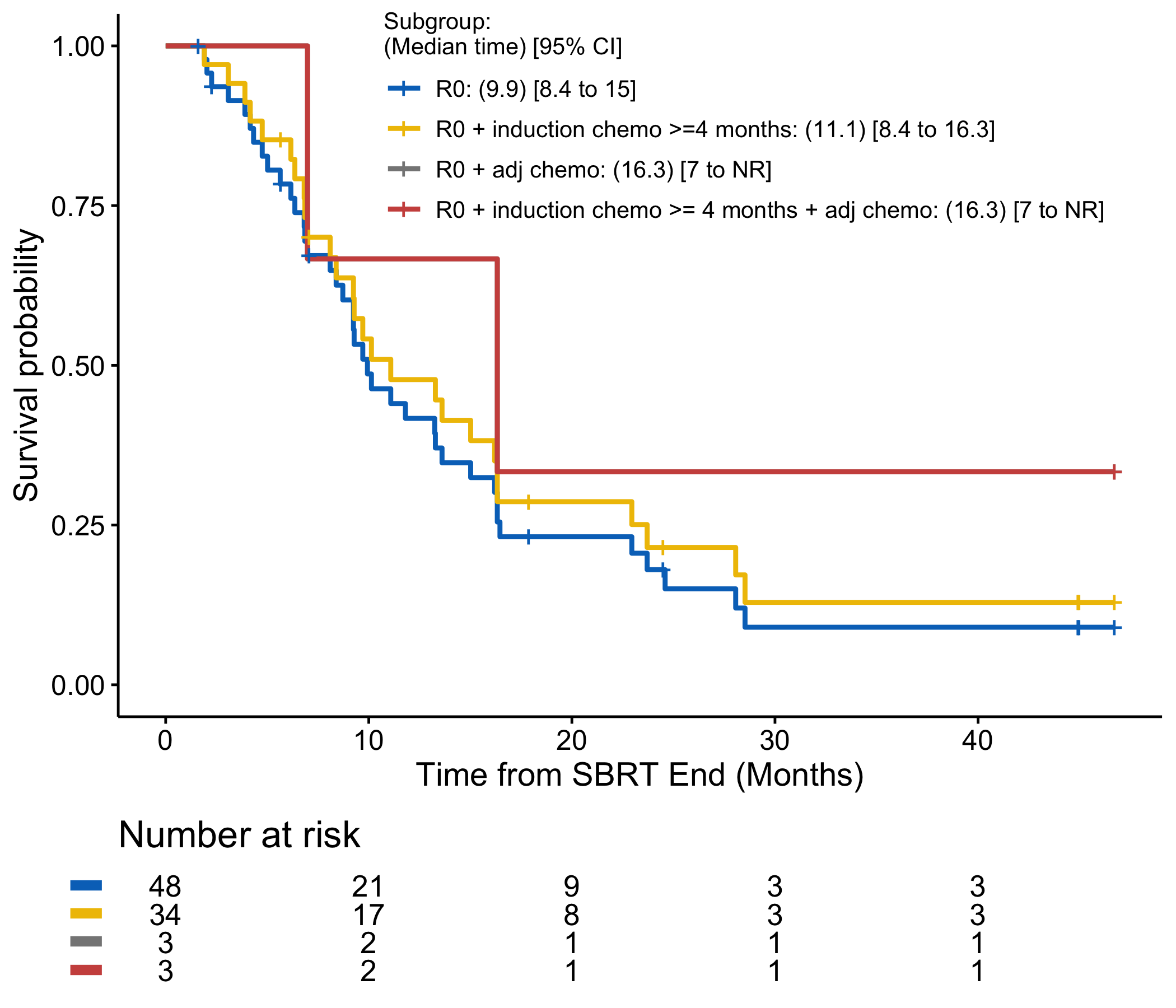

Supplement: Supplementary file 5 — Fig S2 [file CAM4-11-1659-s001.png]
