# Supplementary material for: High local failure rates despite high margin‐negative resection rates in a cohort of borderline resectable and locally advanced pancreatic cancer patients treated with stereotactic body radiation therapy following multi‐agent chemotherapy
Source: Cancer Med. 2022 Feb 10;11(7):1659–68. doi: 10.1002/cam4.4527 (PMC8986142; doi:10.1002/cam4.4527)
Supplement: Supplementary file 4 [file CAM4-11-1659-s002.docx]

Supplementary Table 2: Patterns of Failure for 107 Resected Patients [illegible]

	No Failure	Local Failure
**Margin status**		
R0 > 1mm	27	9
R0 <=1mm	3	4
R1	3	4
**Pathological Complete Response**		
Yes	7	0
No	26	17
**Pathological Nodal Involvement (pN)**		
pN+	9	6
pN0	24	11
**Lymphovascular Invasion (LVI)**		
LVI+	6	2
LVI-	27	12
Indeterminate	0	3
